# The role of histone modifications: from neurodevelopment to neurodiseases

**DOI:** 10.1038/s41392-022-01078-9

**Published:** 2022-07-06

**Authors:** Jisu Park, Kyubin Lee, Kyunghwan Kim, Sun-Ju Yi

**Affiliations:** Department of Biological Sciences and Biotechnology, Chungbuk National University, Cheongju, Chungbuk Republic of Korea

**Keywords:** Epigenetics, Epigenetics in the nervous system, Neurodevelopmental disorders

## Abstract

Epigenetic regulatory mechanisms, including DNA methylation, histone modification, chromatin remodeling, and microRNA expression, play critical roles in cell differentiation and organ development through spatial and temporal gene regulation. Neurogenesis is a sophisticated and complex process by which neural stem cells differentiate into specialized brain cell types at specific times and regions of the brain. A growing body of evidence suggests that epigenetic mechanisms, such as histone modifications, allow the fine-tuning and coordination of spatiotemporal gene expressions during neurogenesis. Aberrant histone modifications contribute to the development of neurodegenerative and neuropsychiatric diseases. Herein, recent progress in understanding histone modifications in regulating embryonic and adult neurogenesis is comprehensively reviewed. The histone modifications implicated in neurodegenerative and neuropsychiatric diseases are also covered, and future directions in this area are provided.

## Introduction

The nervous system allows animals to obtain sensory inputs, integrate data, and move their bodies. It is divided into two main parts in vertebrates, the central nervous system (CNS) and the peripheral nervous system (PNS). The CNS includes the brain and the spinal cord, and the PNS is made up of nerves that branch out from the CNS and extend to all parts of the body. The CNS consists of two main cells, neurons and glial cells, which are generated from the neural stem cells (NSCs).^1^ Neurogenesis is a process by which NSCs transiently become neuronal progenitor cells (NPCs), which differentiate into neurons. Both intrinsic and extrinsic factors coordinate complex signaling pathways that modulate the expression of key regulators that determine NPC proliferation, fate specification, and differentiation.^2–5^ It occurs most actively during embryonic and perinatal stages but also continues throughout life in restricted regions of the brain, the forebrain subventricular zone (SVZ)^6^ and the hippocampal subgranular zone (SGZ).^3,7^ Adult neurogenesis can occur in non-canonical sites, depending on the animal species, age, and physiological/pathological states.^8^ Adult neurogenesis plays an important role in brain homeostasis and functions such as memory and learning. Alterations in adult neurogenesis have been associated with several brain diseases, including neurodegenerative and neuropsychiatric diseases.^9,10^

Neurodegenerative disorders caused by genetic and environmental factors result in loss of integration and function of neurons and even neuronal death. Since the world population is aging rapidly, numerous studies have been conducted to understand the molecular mechanisms in the pathogenesis of neurodegenerative diseases such as Alzheimer’s disease (AD), Parkinson’s disease (PD), and Huntington’s disease (HD), in which aging constitutes a strong risk factor.^3,11–13^ Neuropsychiatric disorders have an early onset (autism and schizophrenia) or a relapsing-remitting course (mood disorder). Although they are associated with alterations in brain function, their apparent etiology is not characterized and the underlying mechanisms are largely unknown. As with neurodegenerative disorders, it is well known that genetic and environmental factors contribute to the pathogenesis of psychiatric disorders.^14,15^

Epigenetics is the study of changes in gene expression that are not attributed to alterations in the DNA sequence in response to intrinsic and extrinsic stimulations. Epigenetic regulatory mechanisms include DNA methylation, histone modification, chromatin remodeling, and microRNA expression. Numerous studies demonstrate that epigenetic regulatory mechanisms are critical in the differentiation of various cells, including bone cells, neurons, cardiomyocytes, and blood cells.^16–20^ Recently, several epigenetic mechanisms in response to extracellular factors have been shown to play a critical role in the control of gene expressions during neurogenesis.^21–27^ In addition, epigenetic dysregulation of neurogenesis contributes to several human diseases, including neurodevelopmental disorders, cognitive impairment, neurodegenerative diseases, and neuropsychiatric diseases.^28^ Dysregulation in adult neurogenesis particularly appears to be a common hallmark in several neurodegenerative and neuropsychiatric diseases.

Herein, recent progress in understanding the role of histone modifications during neurogenesis is reviewed. Furthermore, the perturbation of histone modifications in neurodegenerative and neuropsychiatric disorders to provide insights for new treatment of these neurological disorders is discussed.

## Histone modification

A nucleosome is a fundamental unit of chromatin composed of 147 base pairs of DNA and an octamer of core histone proteins, two copies each of histones H2A, H2B, H3, and H4. Histone proteins are basic and conserved from archaea to humans based on the amino acid sequence.^29,30^ Histone undergoes various post-translational modifications including acylation (e.g., acetylation, benzoylation, butyrylation, crotonylation, glutarylation, lactylation, and so on), ADP-ribosylation, dopaminylation, glycosylation, methylation, phosphorylation, serotonylation, sumoylation, and ubiquitination.^31–36^ Various histone-modifying enzymes have been shown to catalyze the addition or removal of a specific histone modification. Recently, histone tail cleavage by various enzymes is regarded as an alternative modification to remove pre-existing tail modifications but retaining the histone folding region.^37–39^ Histone modifications exert critical effects on the interaction of different histones or histone–DNA interactions. Furthermore, histone modifications provide or remove binding sites for specific protein complexes, including histone-modifying enzymes, chromatin remodeling complexes, and transcription factors. Consequently, histone modifications regulate gene expression by altering the chromatin structure and protein–protein interaction.^37,39–41^ Here we focus on histone modifications and regulation related to neurogenesis and neurological diseases.

### Histone acetylation

Adding an acetyl group to a lysine residue on a histone neutralizes the positive charge on the lysine residue, weakening the interaction between the histone and DNA. Therefore, histone acetylation activates transcription. Histone acetylation is catalyzed by histone acetyltransferases or removed by histone deacetylases.

Histone acetyltransferases (HATs, also called lysine acetyltransferases, KATs) catalyze the transfer of an acetyl group from acetyl coenzyme A to form ε-N-acetyllysine on histone proteins. HATs are classified into several families based on their sequence homology (Table 1).^42–44^ For example, the GCN5-related N-acetyltransferase (GNAT) family includes GCN5/KAT2A and PCAF/KAT2B. The p300/CBP family includes p300/KAT3B and CBP/KAT3A. In addition, TIP60/KAT5, MOZ (MYST3)/KAT6A, MORF(MYST4)/KAT6B, HBO1(MYST2)/KAT7, and MOF(MYST1)/KAT8 are members of the MYST family named after the founding members MOZ, Ybf2/Sas3, Sas2, and Tip60.^42,44^

**Table 1:** Writer, eraser, and biological effect of histone modifications

MOD	Histone	Residue(s)	Biological effect	Writer	Eraser
Ac	H2A	K5	Gene activation	–	–
	H2B	K5, K12, K15, K20	Gene activation	–	–
	H3	K4, K14, K18, K23, K36	Gene activation	–	–
		K9, K27	Gene activation	MOF, p300, PCAF, TIP60	HDAC
		K56	Histone deposition	–	–
	H4	K5, K8, K16	Gene activation	–	–
		K12	Histone deposition	–	–
		K91	Histone deposition	–	–
Me	H3	K4	Gene activation	ASH1L, MLL1-4, SET7/9, SETD2A-B, SMYD	JARID2, KDM1A-B, KDM2B, KDM5A-D, NO66
		K9	Gene repression	GLP, G9a, SETDB1-2, SUV39H1-2	JHDM1D, KDM1A, KDM3A-B, KDM4A-E, KDM7, PHF8,
		K27	Gene repression	EZH1-2, PRC2	JHDM1D, KDM6A-B, KDM7, UTY,
		K36	Gene activation	ASH1L, NSD1-3, SMYD, SET2	KDM2A-B, KDM4A-E, NO66
		K79	Gene activation	DOT1L	PHF8
		R2	Gene activation	PRMT4-7	–
		R8	Gene activation	PRMT2, PRMT5	–
		R17	Gene activation	PRMT4	–
		R26	Gene activation	PRMT4	–
	H4	R3	Gene activation	PRMT1, PRMT3, PRMT5	–
		K20	Gene repression	–	–
P	H2A	S1	Mitosis	MSK1, PKC	–
		T120	Mitosis, gene activation	BUB1, NHK1, VprBP	–
	H2A.X	S139	DNA repair	ATM, ATR, MST1	PP4
	H2B	S14	Apoptosis	MST1	–
	H3	T6	Activation	PKCβ	–
		T3	Mitosis, DNA repair	HASPIN, VRK1	–
		S10	Mitosis, DNA repair	Aurora B, IKKα, MSK1, MSK2, PIM1	PP1
		T11	Mitosis, DNA repair	DLK/ZIP, PRK1	–
		S28	Mitosis, DNA repair	MSK1, MSK2	–
		T45	DNA replication	PKCδ	–
	H4	S1	Mitosis, gene activation	CKII, ScCK1	–
Ub	H2A	K119	Gene repression	BMI/RING1A	–
	H2B	K120	Gene activation	MST1, RNF20/RNF40, ScRad6, UBCH6	–
	H3	K23	Maintenance of DNA methylation	UHRF1	–
Ser	H3	Q5	Gene activation	TGM2	–
La	H3	K18	Gene activation	p300	–
	H4	K12	Gene activation	p300	–
Cr	H3	K9	DNA repair	p300, GCN5, MOF	HDAC1
		K18	Gene activation	p300, GCN5, MOF	–
		K27	Gene activation	GCN5	–
		K4	Gene activation	MOF	HDAC1, SIRT1-3
		K14	Gene activation	GCN5	

*MOD* modification, *Ac* acetylation, *Me* methylation, *P* phosphorylation, *Ub* ubiquitination, *Ser* serotonylation, *La* lactylation, *Cr* crotonylation, – unknown

Histone deacetylation by histone deacetylases (HDACs) induces a closed chromatin structure. Therefore, HDACs are largely associated with transcriptional repression. Eighteen mammalian HDACs have been identified and divided into four classes (class I, II, III, and IV) based on their sequence similarities to yeast HDACs (Table 1).^45–48^ Class I HDACs include HDAC1, 2, 3, and 8; HDAC1 and HDAC2 are involved in transcriptional corepressor complexes (SIN3A, NuRD, and CoREST), and HDAC3 is related to the biological activity of other complexes (SMRT/N-CoR). Class II HDACs are subdivided into class IIa (HDAC4, 5, 7, and 9), and IIb (HDAC6 and 10). Class III HDACs are members of the sirtuin family, SIRT1, 2, 3, 4, 5, 6, and 7. HDAC11 belongs to the class IV HDAC family.^49^

### Histone methylation

Histone methylation, a reversible reaction catalyzed by histone methyltransferases (HMTs) and histone demethylases (HDMs), can occur on the lysine or arginine residues of histone proteins. Lysine residues can be mono-, di-, or tri-methylated, and arginine residues can be asymmetric or symmetric di-methylated or mono-methylated. Histone methylation exerts an important effect on the transcriptional regulation; it can be considered an active mark or a repressive mark of transcription, depending on the methylated residue. Methylation of histone H3 on lysine 4 (H3K4), lysine 36 (H3K36), lysine 79 (H3K79), or arginine 17 (H3R17) is largely involved in transcriptional activation. In contrast, methylation of histone H3 at lysine 9 (H3K9) and lysine 27 (H3K27) or histone H4 on lysine 20 (H4K20) is often related to transcriptional repression^50,51^. Histone lysine methylation is catalyzed by histone lysine methyltransferases (KMTs) and erased by histone lysine demethylases (KDMs). KMTs are divided into two groups: lysine methyltransferases that contain the SET domain and lysine methyltransferases that do not contain the SET domain, such as DOT1L. The SET domain lysine methyltransferases are further divided into several families: H3K4 KMTs (MLL1, MLL2, MLL3, MLL4, SET1A/SETD1A, and SET1B/SETD1B), H3K9 KMTs (SUV39H1, SUV39H2, GLP/EHMT1, G9a/EHMT2, SETDB1/ESET, and SETDB2/CLLL8), H3K27 KMTs (EZH1 and EZH2), and H3K36 KMTs (NSD1, NSD2/WHSC1, ASH1L, and SET2/SETD2) (Table 1). DOT1L includes a seven-beta-strand domain (7βS) instead of the SET domain, which contains a lysine methyltransferase activity toward H3K79.^52,53^ Histone lysine demethylation is achieved by two conserved families of KDMs employing different reaction mechanisms: LSD (lysine-specific demethylase) demethylases and Jumonji C (JmjC) domain-containing demethylases. Members of the LSD family, including LSD1 and LSD2, undergo a FAD-dependent amine oxidation reaction to demethylate mono- and di-methylated lysine residues. LSD1 (also known as KDM1A) is the first identified histone demethylase, which catalyzes the demethylation of H3K4me1, H3K4me2, H3K9me1, and H3K9me2. The second family consists of JmjC domain-containing histone demethylases that use an oxygenase mechanism to remove the methyl group(s) from the specific mono-, di-, and tri-methylated lysine residues. The enzymatic activity of the JmjC domain depends on α-ketoglutarate (α-KG), Fe (II), and molecular oxygen as cofactors in the demethylation reaction.^54^ Approximately 30 JmjC domain-containing proteins have been identified (Table 1).^55,56^

Histone methylation of arginine is catalyzed by protein arginine methyltransferases (PRMTs). PRMTs are classified into three types based on their catalytic activity. Type I PRMTs (PRMT1, 2, 3, 4, 6, and 8) asymmetrically di-methylate arginine residues while Type II PRMTs (PRMT5 and PRMT9) symmetrically di-methylate arginine residues. The Type III enzyme (PRMT7) catalyzes only the mono-methylated arginine formation (Table 1).^57^ Several reports have shown that arginine methylation is a dynamic modification that occurs cyclically, suggesting the existence of an arginine demethylase.^58^ However, the arginine demethylase(s) is yet to be clarified.

### Other histone modifications

Histone phosphorylation was occurred on serine, threonine, and tyrosine. Many kinases and phosphatases regulate histone phosphorylation, which is linked with chromatin condensation, DNA damage response, and transcription (Table 1).^59,60^ Histone can be heavily modified on lysine residue by ubiquitin. Ubiquitination enzymes (E1 activating, E2 conjugating, and E3 ligase enzymes) and deubiquitinating enzymes modulate histone ubiquitination associated with genome stability, cell cycle, and transcription (Table 1).^61^ With advances in mass spectrometry technology, new modifications such as crotonylation (marked by p300), lactylation (marked by p300), and serotonylation (marked by transglutaminase 2, TGM2) are found in neurodevelopment and neurodisease.^62^

## Histone modification in neurogenesis

### Neurogenesis

**Fig. 1:**
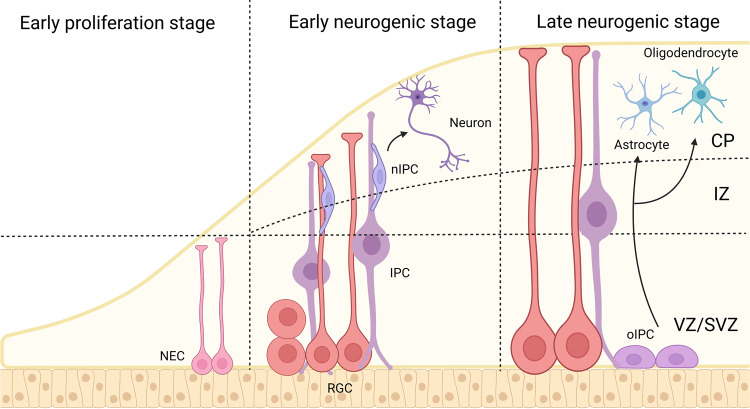
Embryonic neurogenesis. Neuroepithelial cells (NECs) give rise to radial glial cells (RGCs). RGCs divide asymmetrically to produce neuron and neuroglial cells (astrocyte and oligodendrocyte) through neurogenic intermediate progenitor cell (nIPC) and oligogenic intermediate progenitor cell (oIPC), respectively. CP cortical plate, IZ intermediate zone, SVZ subventricular zone, VZ ventricular zone

Neurogenesis, the process by which NSCs differentiate into neurons, occurs throughout life in the embryonic and postnatal/adult brain. Early in embryonic neurogenesis, the neuroepithelial cells (NECs) in the ventricular zone and SVZ in the neural tube develop into radial glial cells (RGCs) with embryonic NSC properties. RGCs undergo symmetric stem cell divisions, expanding the pool. Moreover, NSCs progressively undergo asymmetric cell divisions, allowing self-renewal and the generation of committed daughter cells, such as nascent neurons or neuronal intermediate progenitor cells. Subsequently, the RGCs differentiate into ependymal cells and neurons that migrate to posterior layers along the radial filaments. In the late-stage of development, RGCs produce astrocytes and oligodendrocytes after the formation of the neuron. A small population of residual RGCs develops into adult NSCs responsible for adult neurogenesis (Fig. 1).^3,4,63–65^

**Fig. 2:**
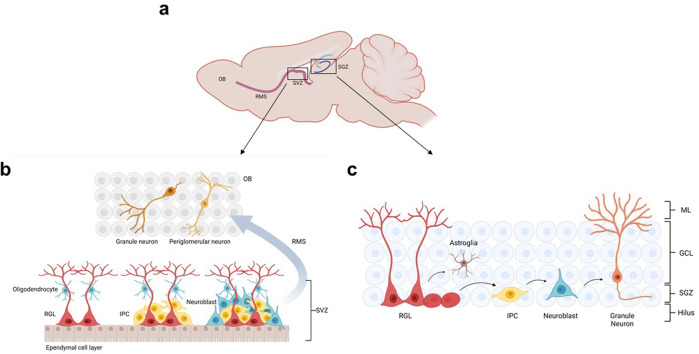
Adult neurogenesis. **a** Adult neurogenesis in the olfactory bulb (SVZ) and hippocampus (SGZ). **b** The process of adult neurogenesis in the olfactory bulb requires a series of steps. RGL activation can generate IPCs that proliferate and differentiate into neuroblast. Some of neuroblasts migrate through the rostal migratory stream (RMS) and become mature and integrate into mature granule or periglomerular neurons in the olfactory bulb (OB). **c** The SGZ niche is composed of RGL, IPC, neuroblast, and granule neuron. Adult neurogenesis in the SGZ undergoes a process similar to that in the SVZ. ML molecular layer, GCL granule cell layer

Unlike embryonic neurogenesis, adult neurogenesis is spatially restricted to two specific locations: the SVZ of the lateral ventricles and the SGZ of the dentate gyrus in the hippocampus (Fig. 2a). Adult NSCs can self-renew and differentiate into neurons, astrocytes, and oligodendrocytes.^66^ Quiescent radial glia-like NSCs (RGLs) can be activated to generate proliferating nonradical transit-amplifying cells (TAPs, also known as intermediated progenitor cells (IPCs)). These cells give rise to immature neurons. In adult SVZ, immature neurons migrate via the rostral migratory stream and develop into granule and periglomerular neurons in the olfactory bulb (Fig. 2b). Newborn cells in the adult SGZ integrate into the granule cell layer of the dentate gyrus and become dentate granule cells (Fig. 2c).^2,67^

Neurogenesis in embryos and adults can be regulated at several levels, including the proliferation of NSCs or progenitors, differentiation and fate determination of progenitor cells, and the survival, maturation, and integration of newborn neurons. Epigenetic mechanisms regulate key regulators’ spatial and temporal expressions critical for NSC/NPC proliferation, differentiation, and fate determination.^23^

### HATs

**Fig. 3:**
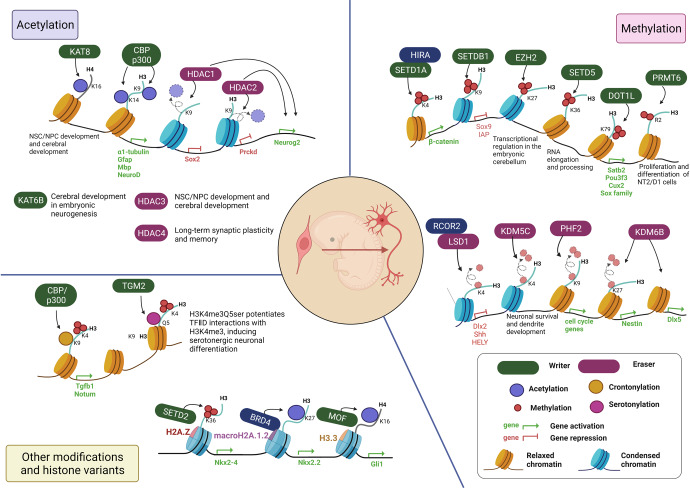
Histone modifications during embryonic neurogenesis. Histone modifications (e.g., acetylation, methylation, crotonylation, and serotonylation) and other epigenetic regulators (e.g., histone variants) are involved in embryonic neurogenesis. Various HATs (p300/CBP, KAT6B, and KAT8) and HDACs (HDAC1-4) are involved in embryonic neural development by regulating gene expression. Changes in histone methylation by writers (SETD1A/1B, SETDB1, EZH2, SETD5, DOT1L, and PRMT6) or erasers (LSD1, KDM5C, PHF2, and KDM6B) control gene expression, promoting embryonic neurogenesis. Other histone modifications (crotonylation and serotonylation) and histone variants (H2A.X, macroH2A1.2, and H3.3) are involved in embryonic neurogenesis

Several HATs regulate neurogenesis (Fig. 3 and Table 2). So far, CBP has been reported to regulate embryonic and adult neurogenesis. Wang et al. showed that CBP haploinsufficiency or CBP knockdown inhibited the differentiation of embryonic cortical precursors into neurons, astrocytes, and oligodendrocytes, coinciding with a decrease in CBP binding and histone acetylation (H3K9/14 acetylation) to neuronal and glial gene promoters such as *α1-tubulin*, *Gfap*, and *Mbp*. The CBP defect caused early cognitive dysfunctions, such as Rubinstein-Taybi syndrome.^68^ CBP/p300 also determines the cell lineage specificity in an activator-dependent manner. CBP/p300 and SMAD1, separately or together, are associated with the transcription factor neurogenin at neural-specific promoters (e.g., NeuroD). These promote embryonic neurogenesis and inhibit astrocyte differentiation.^69^ In addition, histone acetylation by CBP is associated with postnatal neuroplasticity for adaptation to the environment. CBP-deficient mice showed a strong defect in environmental enrichment-induced neurogenesis in the hippocampus, along with reduced histone acetylation (AcH2B, AcH3) at the promoters of environmental enrichment-regulated neurogenesis-related genes such as *Lif*, *Neurog1*, *Dcx*, *Nes*, and *NpY*.^70^

**Table 2:** Role of histone acetyltransferases (HATs) and histone deacetylases (HDACs) during neurogenesis

	Enzyme	Model	Effect on neurogenesis	Histones	Target genes	Refs.
HAT	CBP	Mouse embryonic brain	Embryonic ↑	H3K9/K14ac	*α1-tubulin, Gfap, Mbp*	^68^
	CBP/p300	NSC from mouse embryos	Embryonic ↑		*Neuro D*	^69^
	CBP	Hippocampus of CBP-deficient mice under environmental enrichment	Adult ↑	AcH2B, AcH3	*Lif, Neurog1, Dcx, Nes, NpY*	^70^
	KAT6B	Mutant *KAT6B* mice	Embryonic ↑			^71^
	KAT6B	KAT6B-deficient mice, SVZ cells isolated from KAT6B-deficient mice	Adult ↑			^72^
	KAT8	Cerebrum-specific KO mice using Emx1-Cre	Embryonic ↑	H4K16ac		^73^
HDAC	HDAC1/HDAC2	Conditional KO mice using GFAP-Cre, KO neuronal precursors	Embryonic ↑			^81^
		Conditional KO mice using Emx1-Cre	Embryonic ↑			^82^
		Conditional KO mice using Nestin-Cre	Embryonic ↑			^84^
	HDAC1	Mouse embryonic brain, mouse ESC	Embryonic ↑	H3K9ac	*Sox2*	^85^
		Deletion of HDAC1 by viral infection of Hippocampal NSCs	Adult ↑			^92^
	HDAC2	Conditional KO mice using Nestin-Cre	Embryonic ↑	H3K9ac	*Prkcd*	^84^
		HDAC2 mutant mice, conditional deletion of HDAC2 in adult NSCs	Adult ↑			^90^
	HDAC3	Conditional KO mice using Nestin-Cre	Embryonic ↑			^86^
		Cerebrum-specific KO mice using Emx1-Cre	Embryonic ↑			^87^
	HDAC4	Conditional KO mice using Thy1-cre and Nestin-cre	N/e			^88^
	HDAC5	Hippocampal NSCs from adult rat HCN cells	Adult ↓		*MEF2 target genes*	^96^
	SIRT1	Cortical NPC from mouse embryos	Embryonic ↓	H3K9ac	*Mash1*	^97^
		Neuro 2A (N2a), a mouse neural crest-derived cell line	Embryonic ↓			^99^
		SVZ and Hippocampal Neural Precursors, conditional KO mice using Nestin-Cre	Adult ↓			^98^
	SIRT6	Mice with a heterozygous Sirt6 overexpression	Adult ↑			^100^

↑ Increase, ↓ decrease, *n/e* no effect

KAT6B (MORF, MYST4), a histone acetyltransferase of the MYST family, is essential for neurogenesis. Mice carrying the *Kat6b* gene mutation have developmental defects in embryonic neurogenesis of the cerebral cortex.^71^ Another studies revealed that KAT6B is also involved in adult neurogenesis.^72^ *Kat6b* gene is highly expressed in the neurogenic SVZ, and KAT6B-deficient mice show fewer NSCs and fewer migratory neuroblasts in the rostral migratory stream. Furthermore, NSCs/NPCs isolated from KAT6B-deficient mice showed defects in self-renewal capacity and the ability to differentiate into neurons.

**Table 3:** Role of histone methyltransferases (HMTs) and histone demethylases (HDMs) during neurogenesis

	Enzyme	Model	Effect on neurogenesis	Histones	Target genes	Refs.
HMT	MLL1	Conditional KO mice using GFAP-Cre, NSCs from SVZ	Adult ↑	H3K4me3	*Dlx2*	^103^
	SETD1A	Mouse embryonic NSCs	Embryonic ↑	H3K4me3	*β-catenin*	^104^
	SETD5	Knockdown by in utero electroporation to embryos, NSCs from embryos, Setd5 mutant mice	Embryonic ↑	H3K36me3		^105^
	EZH2	Conditional KO mice using Emx1-Cre	Embryonic ↑			^106^
		Conditional KO mice using Pax7-Cre	Embryonic ↑	H3K27me3		^107^
		Conditional KO mice using hGFAP-CRE, SVZ NSCs	Adult ↑	H3K27me3	*Ink4a/Arf and Olig2*	^108^
		Conditional KO mice using Nestin-Cre	Adult ↑	H3K27me3		^109^
	SETDB1	Conditional KO mice using Emx1-Cre or Nestin-Cre, embryonic NPCs	Embryonic ↑	H3K9me3	*Sox9, IAP*	^110^
	DOT1L	Conditional KO mice using Foxg1-Cre, Emx1-Cre, Nestin-CreER(T2)/R26R-(YFP)	Embryonic ↑	H3K79me2	*Satb2*	^113^
	PRMT6	PRMT6 KO NT2/D1 cells by CRISPR/Cas9 genome editing, constitutive KO mice	Embryonic ↑	H3R2me2a		^114^
HDM	LSD1	Mouse embryonic cortex	Embryonic ↑	H3K4me1	*Dlx2, Shh*	^117^
		Human fetal NSCs	Embryonic ↑	H3K4me2	*HELY*	^119^
	LSD1 + 8a	Rat cortical neuron	Embryonic ↑			^120^
	KDM5B	NSCs isolated from adult SVZ	Adult ↓	H3K4me3	*Reln*	^125^
	KDM5C	Zebra-fish development, rat primary cerebellar granule neurons	Embryonic ↑	H3K4me3		^124^
	KDM6B	Differentiation of mouse embryonic stem cells into NSCs	Embryonic ↑	H3K27me3	*Nestin*	^126^
		Rat neural stem cell	Embryonic ↑		*Dlx5*	^127^
		Targeted Jmjd3-deletion to SVZ NSCs in adult mice by stereotactic injection of the Ad:GFAP-Cre adenovirus into the SVZ	Adult ↑	H3K27me3	*Dlx2*	^128^
	PHF2	Mouse embryonic NSCs, chicken embryo spinal cords	Embryonic ↑	H3K9me2		^129^

↑ Increase, ↓ decrease

A recent study reported that KAT8 (MOF, MYST1), essential for H4K16ac, controls cerebral and NSC/NPC development during embryonic stages. Mutant mice lacking *Kat8*, specifically in the cerebrum, showed cerebral hypoplasia in the neocortex and hippocampus. The study also reported that *KAT8* variants in patients are associated with intellectual disability, seizures, autism, dysmorphisms, and other anomalies, suggesting a link between deficient H4K16 acetylation and intellectual disability.^73^

### HDACs

**Fig. 4:**
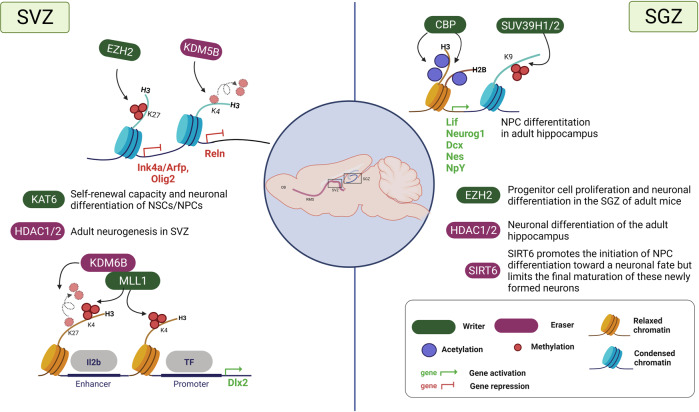
Histone modifications during adult neurogenesis. In SVZ, KAT6 and HDAC1/2 regulate neurogenesis. HMTs (MLL1 and EZH2) and HDMs (KDM5B and KDM6B) regulates gene expression related to neurogenesis. In SGZ, HAT (p300), HDACs (HDAC1/2 and SIRT6), and HMTs (SUV39H1/2 and EZH2) promote neural differentiation by regulating gene expression

In addition to HATs, HDACs are also key regulators in neurogenesis (Figs. 3, 4 and Table 3).^74,75^ HDACs are differentially expressed depending on the cell and tissue types.^76^ HDACs including HDAC1, 2, 4, 5, 6, and 11 and SIRT1 and 6 are known to be enriched in the brain.^77–79^ Of these, HDAC1 and 2 are extensively studied during brain development. HDAC1 and HDAC2 have almost identical genomic organization and a redundant or specific role depending on the stages of brain development.^80,81^ Montgomery et al. generated mice lacking HDAC1 and HDAC2 in the CNS using GFAP-Cre, which is widely expressed in the CNS, including NSCs.^81^ Removal of HDAC1 or HDAC2 has no obvious effect on neuronal development, whereas the removal of HDAC1 and HDAC2 causes major abnormalities in the cortical, hippocampal, and cerebellar development. Additionally, neuronal precursors lacking HDAC1 and HDAC2 do not specifically differentiate into mature neurons and undergo cell death.^81^ Recently, Tang et al. revealed that HDAC1 and HDAC2 redundantly control the spatial positioning of intermediate progenitors (IPs) to form the SVZ in early cortical development.^82^ Developmental stage-specific depletion of both HDAC1 and HDAC2 in radial glial progenitors attenuated Neurog2 expression, resulting in the mispositioning of IPs on the ventricular surface. Although the mechanism underlying HDAC1/2-mediated gene activations needs to be further studied, these results demonstrated that HDAC1 and HDAC2 play an important role as redundant regulators of neuronal differentiation.

In contrast, HDAC1 and HDAC2 also showed distinct cell-type-specific expression patterns. For example, HDAC1 is highly expressed in NSCs/progenitors, but HDAC2 expression is weakly detected in neural progenitors and upregulated in neuroblasts and neurons.^9,83^ To analyze the individual roles of HDAC1 and HDAC2 in neural development, Hagelkruys et al. expressed single alleles of Hdac1 or Hdac2 alleles in the absence of their paralog using Nestin-Cre transgene mice. It was observed that Nestin-Cre-mediated combined deletion of Hdac1 and Hdac2 leads to severely impaired brain development and embryonic lethality in agreement with a previous study^81^ using GFAP-Cre transgene. However, a single allele of Hdac2 but not of Hdac1 in the absence of its paralog prevents brain abnormalities and perinatal lethality. Furthermore, it showed that HDAC2, as a predominant regulator, is recruited to the *Prckd* gene and removes H3K9 acetylation, resulting in repression of *Prckd* expression.^84^ HDAC2 deletion might have different effects on neuronal development due to the different expression patterns of Nestin and GFAP within the NSC and progenitor populations. In addition, the differentiation of HDAC1-deficient mouse ESCs into neurons was repressed, resulting in the modulation of H3K9 acetylation on *Sox2*.^85^ These results propose that HDAC1 and HDAC2 may play specific roles in embryonic neuronal development.

HDAC3, one of the class I HDACs, is also involved in neurogenesis. Conditional knockout of HDAC3 using the Nestin-Cre system showed major abnormalities in brain development.^86^ Furthermore, cerebrum-specific deletion of HDAC3 causes developmental defects in the neocortex and hippocampus and NSC/NPC depletion, indicating that HDAC3 plays an important role in embryonic neuronal development.^87^

Unlike class I HDACs, the conditional deletion of Hdac4 in the CNS using the Thy1-Cre system and the Nestin-Cre system has no obvious defect in the brain architecture or neuronal viability.^88^ Instead, long-term synaptic plasticity and memory were impaired in HDAC4 cKO mice.^89^ These studies proposed that class II HDACs such as HDAC4 might exert their functions in the area of learning and memory.

In agreement with the differential role for class I and II HDACs in embryonic neurogenesis, these HDACs have engaged in adult neurogenesis. In SVZ and DG during adult neurogenesis, HDAC1 is highly expressed in NSCs, and its expression is low or undetected in intermediate progenitor cells and neuroblasts, whereas HDAC2 expression is high in neuroblasts.^90,91^ A recent study showed that HDAC1 is essential for neuronal differentiation of the adult hippocampus^92^, whereas HDAC2 is necessary for the complete differentiation and survival of adult-generated neurons.^90^ HDAC5 has also been implicated in adult neurogenesis.^21^ HDAC5 is known to interact with MEF2 and repress the MEF2 transcriptional activity involved in neurogenesis.^93–95^ Schneider et al. showed that isoxazole, a small neurogenic molecule, triggers a robust neuronal differentiation in adult NSCs via HDAC5 export, which de-represses MEF2-mediated gene expression.^96^

The sirtuin family plays a vital role in determining the fate of neural progenitors. For example, SIRT1 is regulated by a redox state, which influences the fate of mouse embryonic neural progenitor cells. Under oxidative conditions, upregulated SIRT1 interacts with transcription factor HES1, downregulates H3K9ac, and subsequently downregulates pro-neuronal Mash1, resulting in the suppressed proliferation of NPCs and the differentiation of NPCs into the astroglial lineage. Conversely, knockdown of SIRT1 in NPCs induces upregulation of Mash1 and neurogenesis under oxidative conditions.^97^ Another study showed that SIRT1 depletion promotes neuronal differentiation in N2A cells and significantly increases neuronal production in the adult SVZ and the hippocampus.^98,99^ These results indicate that SIRT1 functions as a negative regulator of embryonic and postnatal neurogenesis.^97–99^ In contrast, mice with heterozygous SIRT6 overexpression were used to study the involvement of SIRT6 in adult hippocampal neurogenesis. SIRT6 overexpression did not alter the NPC proliferation in vivo but increased the proportions of young neurons and decreased the proportions of mature neurons without affecting the glial differentiation. Furthermore, it was proposed that SIRT6 promotes the initiation of NPC differentiation toward a neuronal fate but limits the final maturation of these newly formed neurons.^100^

Collectively, members of the HDAC family are differentially involved in both embryonic and adult neurogenesis. Although the direct roles of HDACs in regulating transcription are becoming apparent, an integrated function of HDACs in neurogenesis requires further studies.

### HMTs

Various HMTs are involved in the neurodevelopmental processes (Figs. 3, 4 and Table 2). MLL1 contains a SET domain that catalyzes the methylation of histone H3 at K4. Members of the MLL family are known to recruit H3K27-specific histone demethylases such as KDM6A(UTX) and KDM6B(JMJD3), suggesting cooperation between H3K4 methylation and H3K27 demethylation.^101^ Lim et al. reported that MLL1 is essential for adult neurogenesis in SVZ.^102,103^ MLL1-deficient NSCs in SVZ rarely differentiate into neuronal cells, whereas they can proliferate and differentiate into glial lineages. Chromatin immunoprecipitation demonstrated that *Dlx2*, a key downstream regulator of SVZ neurogenesis, is a direct target of MLL1. In wild-type MLL1 cells, *Dlx2* promoters have high levels of H3K4me3, which activates transcription. In MLL1-deficient SVZ cells, chromatin at *Dlx2* is bivalently marked by H3K4me3 and H3K27me3, inhibiting *Dlx2* expression. These data show that MLL1 functions as a cell fate determinant toward neurogenesis. SETD1A is adopted to the *β-catenin* promoter by histone cell cycle regulator (HIRA), a histone chaperone, and increases H3K4me3 levels, resulting in the induction of *β-catenin* expression in mouse embryonic NSCs. These facilitate neurogenesis of the neocortex.^104^ SETD5 deposits H3K36me3 required for RNA elongation and processing and, ultimately, for proper gene transcription. SETD5 inactivation causes hypo-methylation H3K36 and chromatin states unfavorable for elongation and splicing, altering NSC proliferation, synaptic transmission, and animal behavior.^105^

During the cerebral cortex development, loss of EZH2, an H3K27 methyltransferase, altered the timing of neurogenesis and the relative numbers of different cell types. EZH2 deletion in early embryonic cortical neuronal progenitors induces premature neuronal differentiation, decreasing the number of neurons at birth.^106^ The ablation of EZH2 using Pax7-Cre leads to a decrease in the global H3K27me3 level and transcriptional dysregulation in the embryonic cerebellum. Loss of EZH2 reduced the number of Purkinje cells and increased the number of cerebellar interneurons, and ultimately led to a hypoplastic cerebellum.^107^ EZH2 is also involved in cell proliferation and neuronal lineage specification of adult SVZ NSCs. Loss of EZH2 decreased SVZ NSC neurogenesis in vitro and in vivo. EZH2 is directly recruited to *Ink4a/Arf* and *Olig2* and mediates H3K27 methylation, repressing their expression. Repression of *Ink4a/Arf* or *Olig2* is required for the proliferation of SVZ neurogenesis, respectively.^108^ Consistently, Zhang et al. also showed that EZH2 is essential for progenitor cell proliferation and neuronal differentiation in the SGZ of adult mice.^109^ Taken together, EZH2 is a critical epigenetic regulator in embryonic neurogenesis and adult neurogenesis in both SVZ and SGZ.

Some HMTs showed spatiotemporal expression during cortical neurogenesis, influencing cell fate and localization in different layers. For example, SETDB1 (ESET), a histone H3 Lys9 (H3K9) methyltransferase, is highly expressed during early mouse brain development but downregulated over time. During embryonic neurogenesis, SETDB1 deletion leads to reduced H3K9 trimethylation and dysregulation of gene expressions, such as *Sox9* and *IAP*, resulting in severe impairment of early neurogenesis. SETDB1 deficiency increased neurons in deep layers but decreased neurons in upper layers, suggesting that SETDB1 is required for proper neuronal composition and distribution in all six cortical layers. Furthermore, SETDB1 deficiency increased apoptosis and reduced the proliferation of NPCs isolated from the embryonic brain. These results indicate that SETDB1 as an epigenetic regulator is essential during early brain development.^110^ On the other hand, Suv39h1/2-mediated H3K9 methylation controls the differentiation of NPCs in the adult hippocampus. The number of H3K9me3 foci was significantly increased in NPCs compared to NSCs and then decreased in mature neurons. Pharmacological inhibition of Suv39h1/2 in adult hippocampal progenitors reduced neuronal differentiation while inducing proliferation. Furthermore, knockdown of Suv39h1/2 in adult mouse dentate gyrus impaired neurogenesis, indicating that Suv39h1/2-mediated H3K9me3 is important for adult hippocampal neurogenesis.^111^

Recent studies revealed that H3K79 methylation during cortical development might contribute to a specific layer identity.^112,113^ Franz et al. reported that DOT1L, a histone methyltransferase for H3K79me1/me2/me3, is critical for maintaining the progenitor pool and the proper distribution of deeper layer and upper-layer neurons in the developing cerebral cortex. DOT1L prevents premature differentiation by increasing the expression of genes that regulate asymmetric cell division. Loss of DOT1L caused a decrease in H3K9me2, resulting in decreased transcription of genes expressed in upper-layer neurons (Satb2, Pou3f3, Cux2, and the Sox family). Therefore, DOT1L methylation activity toward H3K79 affects the cell cycle and activates transcription related to upper-layer identity in early progenitors.^113^

In addition to histone methylation on lysine residues, histone methylation on arginine residues is involved in the regulation of neurogenesis. For example, asymmetric di-methylation of histone H3 at arginine 2 (H3R2me2a) is occurred in a PRMT6-dependent manner primarily at the promoter and enhancer sites of genes in the human embryonal carcinoma cell line NT2/D1.^114^ In particular, promoter-associated H3R2me2a counteracted KMT2A-mediated H3K4me3, resulting in reducing the transcription of critical pluripotency genes. However, enhancer-associated H3R2me2a increases the differentiation-associated genes by inducing H3K4me1 (KMT2D) and H3K27ac (p300). That is, PRMT6 is essential for the differentiation and proliferation of NT2/D1 cells and neural precursor cells in the developing mouse cortex.

### HDMs

Like HMTs, several HDMs play key roles in the neurodevelopmental processes (Figs. 3, 4 and Table 2). As mentioned above, LSD1 (also known as KDM1A) is the first enzyme identified as a histone demethylase and has been reported to mediate the demethylation of H3K4me1/2 and H3K9me1/2, leading to repression and stimulating transcription, respectively. Numerous studies have reported the role and function of LSD1 in brain development. LSD1 is required for the maintenance of neural progenitor/precursor cells.^115–117^ Fuentes et al. demonstrated that LSD1 as a component of the CoREST complex plays an important role in neuronal differentiation, migration, and morphology during mouse cortical development.^116^ LSD1-RCOR2 (CoREST2) are recruited at the promoter of the *Dlx2* and *Shh* genes, which inhibit the Shh pathway in the developing neocortex, thereby inducing cortical neurogenesis.^117^ Another study showed that LSD1 mediates H3K4me2 demethylation on the *HEYL* promoter and blocks *HEYL* gene expression that inhibits human fetal NSC neuronal differentiation.^118,119^ In contrast to the repressive function of LSD1 on transcription, Zhang et al. showed that LSD1 increases atrophin1 expression by controlling the histone methylation status in the enhancer region, which is important for NPC maintenance.^115^ Zibetti et al. found that alternatively splicing an LSD1 transcript produces four full-length isoforms, and the expression of neuron-specific LSD1 isoforms is regulated during brain development and contributes to neurite morphogenesis during early cortical development.^120^ LSD1 + 8a, an isoform of LSD1, mediates H3K9me2 demethylation, but not H3K4me3 demethylation, along with supervillin (SVIL). The LSD1 + 8a/SVIL-containing complex plays a crucial role in the neuronal gene expression and neuronal differentiation in human neuroblastoma cells.^121^

KDM5B catalyzes the removal of methyl groups on H3K4me3, which is primarily associated with transcriptional repression. KDM5B depletion reduces proliferation but promotes the migration of NSCs isolated from adult SVZ. Whole-genome expression screening showed that KDM5B depletion caused widespread transcriptional changes, including notably, the upregulation of the secreted extracellular matrix glycoprotein reelin (Reln). ChIP assays revealed that KDM5B is recruited to proximal promoter regions of *Reln* and removes H3K4me3, resulting in decreased *Reln* transcription. These results indicate that KDM5B reduces the neurogenesis of adult SVZ NSCs.^122^

KDM5C (SMCX, JARID1C) is one of the most frequently mutated genes in X-linked mental retardation (XLMR).^123^ Iwase et al. reported that KDM5C inverts H3K4me3 to di- and mono- but not unmethylated products. Depletion studies in zebrafish and primary mammalian neurons demonstrated that KDM5C plays a role in neuronal survival and dendrite development.^124^ Several point mutations from XLMR patients reduce KDM5C demethylase activity and suppress neurite growth in Neuro2a cells. These results suggest that KDM5C is involved in neuron differentiation and the pathogenesis of XLMR.^124,125^

KDM6B (also known as JMJD3), an H3K27-specific demethylase, functions as a crucial activator of neurogenesis. KDM6B is required for neuronal commitment during the differentiation of embryonic stem cells into NSCs. KDM6B controls major regulators and markers of neurogenesis such as Nestin by regulating the dynamics of H3K27me3.^126^ Furthermore, the KDM6B expression and its recruitment to the *Dlx5* promoter are regulated in NSC differentiation in response to retinoic acid.^127^ KDM6B also induces neurogenesis from NSCs in adult SVZ. Park et al. showed that *Kdm6b* deletion targeted to SVZ NSCs in both developing and adult mice impairs neuronal differentiation. KDM6B regulates the neurogenic gene expression via interaction at not only promoter regions but also neurogenic enhancer elements. In KDM6B-deleted SVZ cells, H3K27me3 levels are increased at I1/2b, an enhancer regulating the expression of *Dlx2*, which leads to *Dlx2* gene repression.^128^ Considering the previous finding that MLL1 deficiency in SVZ cells increases H3K27me3^103^, Park et al. studied a relationship between MLL1 and KDM6B in the regulation of *Dlx2* expression. In differentiating SVZ cells, MLL1 was localized at both the I12b enhancer and promoter of *Dlx2*. In MLL1-deleted NSCs, KDM6B was not recruited at I12b, but, in KDM6B-deleted cells, MLL1 remained enriched at I12b. Together, these results suggest that MLL1-dependent localization of KDM6B at I12b is required for lifelong neurogenesis.

PHF2 is a member of the KDM7 family that demethylates H3K9me2. Recently, the PHF2 was discovered to play an essential role in NSC biology and early neurogenesis in the chicken neural tube. In mouse embryonic NSCs, PHF2 binds to cell cycle gene promoters and mediates H3K9me2 demethylation, which regulates cell cycle gene transcription. PHF2 depletion in chicken embryo spinal cords reduced early neurogenesis by controlling the progenitor proliferation.^129^

### Other histone modifications

In addition to histone acetylation and methylation, various histone modifications are associated with neurogenesis (Fig. 3). Recent studies have shown that histone lysine is crotonylated by p300/CBP.^130,131^ Histone lysine crotonylation (Kcr) activates gene expression and influences development and disease processes.^132–134^ Dai et al. showed that H3K9cr was significantly enriched at active promoters co-marked by H3K4me3 and H3K27ac in embryonic forebrain at E13.5. In particular, an increase in histone Kcr and a decrease in H3K27me3 were observed in the bivalent promoters (e.g., *Tgfb1* and *Notum*). Elevated histone Kcr promotes NSC/NPC cell fate decision and accompanies embryonic neurogenesis.^135^

Serotonin is not only a well-known neurotransmitter that modulates neuronal activity, but it can be also covalently bound to glutamine, called serotonylation.^136,137^ Farrelly et al. revealed that transglutaminase 2 (TGM2) directly attaches serotonin to the glutamine residue at the fifth position of H3 (H3Q5ser). Although TGM2 serotonylated both H3K4me0- or H3K4me3-marked nucleosome, producing H3Q5ser or H3K4me3Q5ser, the dual mark (i.e., H3K4me3Q5ser) was only identified in brain. H3K4me3Q5ser was enriched in euchromatin and potentiated TFIID interactions with H3K4me3, which induced serotonergic neuronal differentiation.^34^

### Interplay of histone variants and histone modifications

Histone variants are nonallelic isoforms of canonical core histones, and their specific deposition onto chromatin is involved in transcriptional regulation.^138^ Deposited histone variants cooperate with histone-modifying enzymes to regulate gene expression. Recent studies showed that the association of histone variants with histone-modifying enzymes plays a key role in neurogenesis (Fig. 3).

In the histone H2A family, there are four major replacement H2A variants: H2A.Bbd, H2A.X, H2A.Z, and macroH2A. H2A.Z (60% identity with H2A) preferentially localizes at transcription start sites (TSSs) and regulates gene expression via the recruitment of various H2A.Z interactors.^139,140^ Shen et al. showed that brain-specific H2A.Z deletion increased NPC proliferation but decreased neural differentiation. H2A.Z regulates embryonic neurogenesis by specifically interacting with SETD2 in the *Nkx2-4* promoter region to promote H3K36me3 and *Nkx2-4* expression.^141^ MacroH2A has a unique tripartite structure: an H2A-like histone domain (65% identity of H2A), a linker region, and a globular macro domain. It has two isoforms known as macroH2A1 and macroH2A2. While macroH2A is important for X-chromosome inactivation and autosomal gene silencing, macroH2A exerts both positive and negative effects on gene transcription in a context- and isoform-dependent manner.^142^ Similar to H2A.Z, macroH2A is thought to regulate gene expression by cooperating with transcriptional machineries.^143^ Ma et al. found that deletion of macroH2A1.2, one of macroH2A1 splice variants, enhanced NPC proliferation and reduced NPC differentiation, indicating that macroH2A1.2 is required for embryonic neurogenesis. MacroH2A1.2 physically associates with BRD4, thereby increasing H3K27ac levels and *Nkx2.2* expression.^144^ Last, H2A.X differs from canonical H2A in that it has SQ[E/D]Φ motif (where Φ indicates a hydrophobic amino acid) in the C-terminus, which can be phosphorylated in response to DNA damage or cell cycle progression. Recent studies showed that H2A.X phosphorylation (γH2AX) was expressed in the mouse brain from embryonic life to senescence. γH2AX is observed during embryonic and adult neurogenesis and appears to be involved in neuronal proliferation and differentiation.^145,146^ The exact role of γH2AX during neurogenesis needs to be examined.

H3.3 is the predominant form of histone H3 variants that differs by four to five amino acids from canonical histone H3. It was reported that H3.3 is required for embryonic NSCs proliferation. Furthermore, knockdown of H3.3 causes the abnormal neuronal development. H3.3 interacts with MOF, which increases H4K16ac levels in the Gli1 promoter and activates Gli1 expression.^147^

### Corepressor complexes including histone-modifying enzymes

Most histone-modifying enzymes often function as a part of multiprotein complexes to regulate neurogenesis. There are three best-studied corepressor complexes as regulators of neurogenesis: CoREST (corepressor for element-1-silencing transcription factor), NuRD (nucleosome remodeling and deacetylation), and Sin3.^117,148–151^ The main components of the CoREST complex are HDAC1/2, LSD1, CoREST1/2/3, ZNF217, and PHF21A. As mentioned, RCOR2 (CoREST2) plays a key role in neurogenesis during mouse brain development.^116,117^ Deletion of RCOR2 (CoREST2) results in the decrease of NPC proliferation, neuron population, neocortex thickness, and brain size. RCOR2 inhibits the Shh signaling pathway by recruiting the LSD1 complex and binding directly to the *Dlx2* and *Shh* genes, resulting in H3K4me1 demethylation.^117^ Recently, the cortex-specific PHD finger protein PHF21B (plant-homeodomain finger protein 21B), originally known as a tumor suppressor^152^, was highly expressed in the neurogenic phase of cortical development and gradually decreased as the astrogliogenic phase started. PHF21B depletion in utero electroporation assay shows impaired neurogenesis. PHF21B recruits HDAC2 and LSD1 and causes loss of H3K4me1 and H3K27ac to the promoters of cell cycle genes, resulting in cell cycle exit during neurogenesis.^153^ The MiDAC complex, a histone deacetylase complex, consists of HDAC1/2, two scaffold proteins ELMSAN1 (also known as MIDEAS) and DNTTIP1. CRISPR/Cas9-mediated deletions of *Dnttip1* and *Elmsan1* in mESCs affect neuronal maturation and/or differentiation. MiDAC increases gene expression of pro-neural genes such as *Slit3* and *Ntn1* by removing the repressive H4K20ac on promoters and enhancers. In contrast, MiDAC reduces gene expression of negative regulators of neurogenesis, *Spry4*, and *Id1* by reducing the active H3K27ac.^154^

## Histone modification in neurodegenerative disorders

### Alzheimer’s disease

Alzheimer’s disease, a chronic neurodegenerative disease, is the most common cause of dementia in the elderly. AD was initially described by Dr. Alois Alzheimer in 1906.^155^ Both the β-amyloid (Aβ) plaque deposition and neurofibrillary tangles associated with hyperphosphorylated tau proteins are specific features of AD. The Aβ peptide is the product of amyloid precursor protein (APP) cleavage by β-secretase (BACE1) and γ-secretase (presenilin, nicastrin, anterior pharynx-defective 1 and presenilin enhancer-2). Overproduction of Aβ causes it to self-assemble into oligomers and forms toxic Aβ plaques.^156^ Mutations in genes such as *APP*, *presenilin* (*PSEN1*), *PSEN2*, and *apolipoprotein E (APOE)* contribute to Aβ oligomer toxicity and Aβ peptide deposition, causing familial AD. The main component of the neurofibrillary tangles is an abnormally hyperphosphorylated and aggregated form of tau. Tau (MAPT), a microtubule-associated protein in axons, promotes assembly and stability of microtubules and vesicle transport. In AD, hyperphosphorylated tau is insoluble, lacks affinity for microtubules, and self-aggregates into structures of paired helical filament. Since cellular clearance systems begin to fail during aging, defective clearance of Aβ and tau leads to the accumulation of Aβ and tau aggregates in the AD brain. These Aβ plaque deposition and neurofibrillary tangles cause neurodegeneration and, in turn, synaptic injury and neurogenesis defects, contributing to cognitive dysfunction.^10,157–159^ Although genetic and environmental factors contribute to AD, the strong risk factor is aging, which alters the chromatin structure.^13,160,161^

### Dysregulation of histone modifications in Alzheimer’s disease

**Fig. 5:**
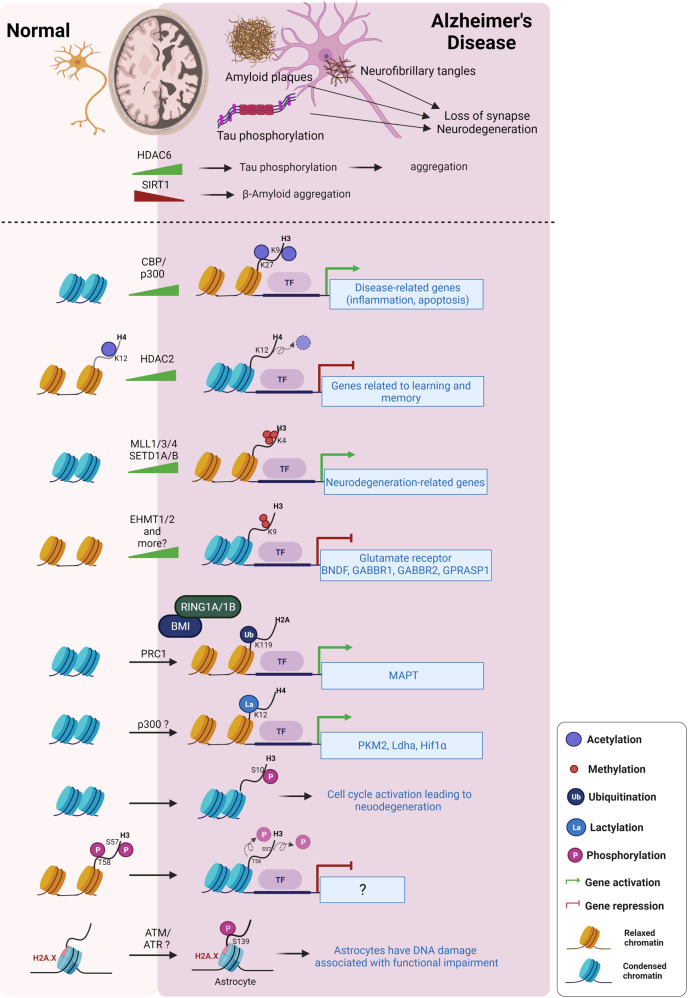
Dysregulation of histone-modifying enzymes and histone modifications in Alzheimer’s disease (AD). Histone acetylation such as H3K9ac and H3K27ac is globally increased due to the increase of CBP/p300 expression. In contrast, acetylation of H3K12 is reduced by the increased HDACs (e.g., HDAC2). In AD, HMTs responsible for H3K4me3 and H3K9me2 are overexpressed: SETD1A/1B and MLL3/4 for H3K4me3, GLP/G9a (EHMT1/2) for H3K9me2. These affect the levels of H3K4me3 and H3K9me2 on a global and gene-specific level, thereby altering gene expression. H2A ubiquitination (H2AK119ub by PRC1), H3 lactylation (H3K12la by p300), H2A.X phosphorylation (H2A.XS139p) are increased in AD. SIRT1 downregulation in AD leads to the elevated Aβ levels and their aggregation. HDAC6 increase in AD stimulates tau phosphorylation, thereby facilitating tau aggregation

Dysregulation of histone acetylation is related to memory decline and AD (Fig. 5).^162^ Histone acetyltransferase activity of CBP (KAT3A) is required for neurogenesis and memory consolidation in rodents.^163–167^ Several studies suggested that an increase in CBP expression is considered a valid therapeutic approach for AD. Conditional double-deletion mice lacking two presenilins (presenilin1 and 2) in the postnatal forebrain show impaired hippocampal memory and synaptic plasticity and subsequently develop synaptic, dendritic, and neuronal degeneration based on their age. Saura et al. showed that the deletion of presenilin1/2 reduced CBP levels, which decreased the transcription of CREB/CBP target genes such as c-fos and BDNF.^168^ A triple-transgenic model of AD (3xTg-AD) harboring three mutant genes (APP, PSEN1, and tau) shows plaque and tangle pathologies, and synaptic dysfunction and impaired neurogenesis.^169,170^ CREB activation and phosphorylation are impaired in the 3xTg-AD mice. CBP gene transfer restores the CREB activity, which increases BDNF levels and improves learning and memory deficits in 3xTg-AD.^171^ Another AD model mice-harboring the familial AD-linked APPswe/PS1ΔE9 mutations reduced levels of phosphorylated CREB, and its cofactors CBP and p300.^172^ A recent study showed that the p300 acetyltransferase activity is increased in the hippocampal area of patients with AD.^173^ Transcriptomic analysis of the lateral temporal lobe of patients with AD against the young and elderly people without dementia revealed elevated levels of CBP/p300 and TRAPP (a component of the SAGA complex), which mediate the deposition of H3K27ac and H3K9ac in the disease-related genes.^174^ These discrepancies may be explained by methodological differences (human sample versus transgenic mice) or differential risk factors between humans and mice, but further study is needed to clarify these issues.

Since HDACs are involved in neurogenesis, brain function, and neurodegenerative diseases, they have become potential therapeutic targets for treating neurodegenerative diseases such as AD and PD.^175,176^ HDAC2, neither HDAC1 nor HDAC3, is increased by AD-related neurotoxic stimuli in vitro, in two models of neurodegeneration in mice and in patients with AD. HDAC2 binds to and reduces histone acetylation of genes important for learning and memory and suppresses their expression.^177^ Knockdown of upregulated HDAC2 increased H4K12ac, leading to de-repression of these genes.^178^ HDAC6, a class II member of the HDAC superfamily, interacts with tau, thereby increasing tau phosphorylation (depending on HDAC activity) and accumulation, and the protein level of HDAC6 in the AD brain is significantly increased compared with the normal brain.^179^ The reduction of endogenous HDAC6 levels rescues Aβ-induced impairment of mitochondrial trafficking and memory impairment in a mouse model for AD.^180^ Sirtuins such as SIRT1 and SIRT2 downregulate Aβ expression. Because the decreased levels of SIRT1 expression in AD lead to the loss of a protective role, they have become potential pharmacological targets to treat AD.^181–183^

Several HMTs were elevated in patients with AD (Fig. 5).^162,184–186^ H3K4me3 methyltransferases such as MLL3 (KMT2C), MLL4 (KMT2D), SETD1A, and SETD1B were significantly elevated in patients with AD, compared to control subjects. In mutant tau transgenic mice, MLL1 (KMT2A) and SETD1B were significantly higher, compared with WT mice. Furthermore, H3K4me3 was significantly increased in the nuclear fraction of the prefrontal cortex lysates from patients with AD and human P301S tau transgenic mice (P301S tau mice), while no significant changes were found in the level of repressive H3K27me3 or enhancer H3K4me1. These results indicate that upregulation of H3K4 HMT results in the elevated level of H3K4me3 in patients with AD and in P301S tau mice.^187^ Repressive mark H3K9me2 and histone methyltransferases EHMT1 and EHMT2 were significantly elevated in the late-stage familial AD mouse model and in patients with AD. Specifically, H3K9me2 in glutamate receptor genes was increased in the prefrontal cortex of the aged familial AD mouse model, which was linked to reduced transcriptions. EHMT1/2 depletion causes decreased H3K9me2 and recovery of glutamate receptor expression and excitatory synaptic function in the prefrontal cortex and hippocampus.^188^

In addition to histone acetylation and methylation, other histone modifications including ubiquitination, phosphorylation, and lactylation are associated with AD. BMI1, a component of PRC1 that promotes chromatin compaction and gene silencing through E3 ligase activity mediated by RING1A/B (H2AK119ub), is silenced in late-onset sporadic AD brain (cortical neuron). Bmi1 knockout in human post-mitotic neurons induced amyloid-beta peptide secretion and deposition, p-Tau accumulation, and neurodegeneration. These results indicate that PRC1-mediated H2Aub is required for repressing MAPT transcription.^189^ Mass spectrometry analysis using frontal cortex from human donors with AD showed that H2BK120 ubiquitination was increased in AD, but further studies will be needed.^185^

Several studies showed that histone phosphorylation is related to AD. In frontal cortex from AD patients, H3 phosphorylation is globally increased.^190^ Another study revealed that H3S10p was significantly increased in hippocampal neurons in AD. However, this phosphorylation is restricted to cytoplasm rather than nucleus, implying that the aberrant localization of H3S10p is associated with neuronal degeneration.^191^ In contrast, H3S57p and/or H3T58p were significantly decreased in a mouse model of rapid amyloid deposition (5XFAD) that produced high levels of Aβ. Decreased phosphorylation of these residues induce heterochromatin, leading to decreased gene expression.^192^ Myung et al. reported that H2A.X phosphorylation was increased in astrocyte of AD, suggesting that astrocytes have DNA damage associated with functional impairment.^193^

Recent study revealed lysine lactylation (Kla) as a new type of histone mark. Glycolysis-derived lactate was used for Kla.^194^ Pan et al. found that lactate levels are increased in hippocampus of 5XFAD mice. H4K12la levels were increased in Aβ plaque-adjacent microglia. Genome-wide analysis showed that H4K12la is enriched at the promoters of glycolytic genes (*PKM2*, *Ldha*, and *Hif-1α*) and activates transcription, thereby increasing glycolytic activity. This positive feedback loop triggers microglial-mediated neuroinflammation, resulting in exacerbating pathogenesis of AD.^195^

### Genome-wide alteration of histone modifications and chromatin accessibility of Alzheimer’s disease

Recent next-generation sequencing technologies, such as ChIP-seq, ATAC-seq, and RNA-seq, have enabled researchers to study genome-wide chromatin dynamics and gene regulation. Numerous studies tried to characterize the relationship between histone modifications and genome-wide chromatin dynamics because AD is associated with dysregulation of histone acetylation and methylation (Fig. 5).^184,185^

H3K9ac is associated with transcription activation and open chromatin. Recently, a genome-wide study was carried out on the dorsolateral prefrontal cortex of 669 elderly human subjects using the H3K9ac mark and neuropathological examination to distinguish between amyloid-beta- and tau-related epigenomic changes. Unlike amyloid-β, tau tangles exert a broad effect on the epigenome, affecting 5990 of the 26,384 H3K9ac domains. Furthermore, the tau-related changes of H3K9ac are greater in open chromatin compartments than in closed chromatin compartments, indicating that tau leads to large-scale changes in histone acetylation and chromatin rearrangement.^196^

H4K16ac, an active mark, is localized to both enhancers and promoters and is related to aging and DNA damage.^11^ Nativio et al. compared the genome-wide enrichment of H4K16ac in the lateral temporal lobe of patients with AD with both age-matched and younger individuals without dementia to investigate crucial mechanisms for AD pathology. Normal aging is associated with a significant increase in the H4K16ac peaks. However, H4K16ac is reduced in patients with AD, suggesting that normal age-related changes in brain H4K16ac are altered in AD. Moreover, AD-associated H4K16ac peaks are implicated with AD-associated single-nucleotide polymorphisms and quantitative trail loci detected specifically in AD studies. Alteration of H4K16ac in normal aging leads to the epigenetic landscape of AD.^197^

H3K27ac is a transcription activation mark. Marzi et al. performed ChIP-seq to quantify H3K27ac levels across the genome in postmortem entorhinal cortex samples from AD cases.^198^ The differential H3K27ac peaks among AD cases and controls were found with significant enrichment of hypoacetylated AD-associated peaks compared with hyperacetylated AD-associated peaks. Differential H3K27ac peaks are enriched in regulatory regions annotated to genes previously implicated in tau and amyloid neuropathology such as *PSEN1*, *PSEN2*, and *MAPT*, and in regions containing variants associated with late-onset sporadic AD.

Recently, considering the complexity of aging and neurodegeneration, Nativio et al. integrated transcriptomic, proteomic, and epigenomic analyses of postmortem human brains (lateral temporal robe) to identify epigenetic alterations associated with AD compared with older and younger controls.^174^ First, previous RNA-seq data were analyzed, presenting the upregulation of the chromatin genes such as CBP, p300, and TRRAP (a subunit of the SAGA–ATAC complex) in the temporal lobe of AD brains as mentioned earlier. Proteomic analysis in comparing AD and old brains revealed changes in both histone methylation and acetylation. As histone methylation is increased or decreased, histone acetylation such as H3K27ac and H3K9ac is elevated in AD. Genome-wide analysis using ChIP-seq revealed AD-specific gains of H3K27ac and H3K9ac in genes that were associated with functional categories related to transcription and chromatin modification. In addition, using fly AD models, it was reported that an increase in H3K27ac and H3K9ac accelerates amyloid-β42 (Aβ42)-induced neurodegeneration in vivo. These results indicate that the H3K27ac and H3K9ac gains in AD dysregulate transcription and chromatin–gene feedback loops, affecting pathways associated with AD.

H3K4me3, a permissive histone mark, is abundant around TSSs. P301S tau mice, a model of AD-associated tauopathy, show significantly elevated levels of H3K4me3 in the prefrontal cortex. Integration analysis of H3K4me3 ChIP-seq and RNA-seq identified genes, which show upregulated expression and increased H3K4me3 occupancy. These genes include *Sgk1*, *Egr1*, *Kcnk1*, *Ddit4*, *Per1*, and *Nfkbia*. Administration of a specific Sgk1 inhibitor reduces a hyperphosphorylated tau protein, restores glutamatergic synaptic function, and ameliorates memory deficits in AD mice.^187^

H3K9me2 is associated with transcriptional repression. The global H3K9me2 and EHMT1/2 were upregulated in the prefrontal cortex in the late-stage familial AD mouse model and in patients with AD.^188^ H3K9me2 ChIP-seq revealed that the familial AD mouse model shows the genome-wide enhanced enrichment of H3K9me2 around the TSS across the genome. H3K9me2 enrichment around the TSS of *Grin2a/NR2A* and *Grin2b/NR2B*, glutamate receptor genes, is significantly increased in the familial AD mouse model, which is reversed by EHMT1/2 inhibition. These results demonstrate that reduced glutamate receptors expression in AD could be due to the dysregulation of H3K9me2. In contrast, a widespread loss of heterochromatin containing H3K9me2 and HP1 has been observed in the head of tau transgenic Drosophila and in motor neurons of tau transgenic mice and in the hippocampal neurons of patients with AD.^199^ In Drosophila tauopathy models, wild-type and mutated tau reduced H3K9me2, upregulating the expression of genes such as *Nvd*, *Ir41a*, and *Ago3* that are normally silenced or expressed at low levels in fly. The RNAi-mediated reduction of Ago3 levels in tau transgenic Drosophila ameliorates the locomotor defects of tau transgenic Drosophila but does not affect transgenic tau or tau phosphorylation levels. These data indicate that tau-induced loss of heterochromatin is involved in neurodegeneration.

H3K9me3, a repressive mark, is associated with heterochromatin. Lee et al. performed an integrated analysis of H3K9me3 ChIP‐seq and mRNA‐seq on cortex tissues from brain samples obtained from patients with AD and healthy subjects.^200^ They found that H3K9me3 is marked differentially and that the transcriptome is differentially regulated between AD and normal brains. Furthermore, H3K9me3-enriched genes inversely correlated with their mRNA expression levels in AD. H3K9me3‐landscaped genes in AD are associated with synaptic transmission, neuronal differentiation, and cell motility, which includes *BDNF, GABBR1, GABBR2*, and *GPRASP1*.

AD-associated chromatin accessibility was investigated via ATAC-seq in the APPswe/PS1dE9 (APP/PS1) mouse model.^201^ Chromatin accessibility around the TSS regions of APP/PS1 mice was higher than those of WT mice, indicating that chromatin accessibility was altered in the APP/PS1 mice. Moreover, AD-increased chromatin-accessible regions contain binding motifs of numerous transcription factors, including Olig2, NeuroD1, TCF4, and NeuroG2. Several genes involved in AD development were found to have significantly higher chromatin accessibility and gene expression in APP/PS1 mice, including *Sele*, *Clec7a*, *Cst7*, and *Ccr6*. The transcription active marks, H3K4me3 and H3K27ac, are highly enriched in the promoter regions of these genes. These results suggest that alterations in chromatin accessibility related to the histone marks H3K4me3 and H3K27ac could be attributed to the enrichment of open chromatin regions with transcription factors motif, resulting in the upregulation of genes involved in AD pathogenesis.

### Parkinson’s disease

Parkinson’s disease, the second most common neurodegenerative disease in the world, was first described by James Parkinson, a British physician in 1817.^202^ Motor symptoms of PD include tremor, postural instability, muscle stiffness, and bradykinesia. Nonmotor symptoms such as depression and cognitive impairments also occur in PD patients.^10,203^ PD is characterized by a loss of dopaminergic neurons in the substantia nigra and the accumulation of intracellular protein inclusions called Lewy body, composed of alpha-synuclein.^202,204^ Although the normal neuronal function of α-synuclein protein is not fully understood, it plays a role in synaptic vesicle dynamics, mitochondrial function, and intracellular trafficking. The neurotoxic accumulation and aggregation of α-synuclein can be triggered by relative overproduction of the protein, the presence of mutations that could increase its misfolding and oligomerization, and defects in the degradation of native or misfolded α-synuclein. Age-related deficiencies in proteolytic clearance mechanisms could play an important role in α-synuclein accumulation. Genetic abnormalities and environmental factors could promote these processes.^205–207^ Hereditary forms of PD only represent 10%–15% of all cases. The genes implicated in the onset of PD (PARK genes) currently comprise 20 genes including *SNCA* (encoding α-synuclein), *LRRK2* (encoding leucine-rich repeat kinase 2, LRRK2), and *VPS35* (encoding vacuolar protein sorting 35).^208,209^ In addition to genetic abnormalities, toxins such as 1-methyl-4-phenyl-1,2,3,6-tetrahydropyridine (MPTP), 6-hydroxydopamine (6-OHDA), paraquat, rotenone, and dieldrin caused the loss of dopaminergic neurons and increased the expression and aggregation of α-synuclein, which are also used to investigate PD pathogenesis.^204,210,211^

### Dysregulation of histone acetylation in Parkinson’s disease

**Fig. 6:**
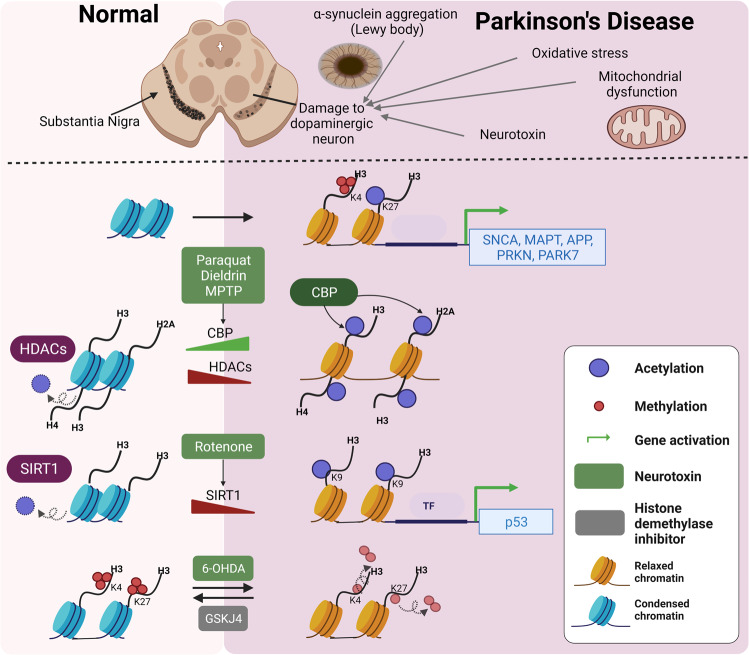
Dysregulation of histone-modifying enzymes and histone modifications in Parkinson’s disease (PD). Several neurotoxins induce H3 and H4 acetylation together with changing the expression of HATs and HDACs. For example, Paraquat and MPTP increase H3 and H4 acetylation. Dieldrin induces H3K14ac by increasing CBP expression. Rotenone reduces SirT1 expression levels, thereby increasing H3K9ac. Histone methylation is also dysregulated in PD. PD-related neurotoxin such as 6-OHDA reduces H3K4me3 and H3K27me3. Furthermore, H3K4me3 at the SNCA promoter is increased in PD patient

Neurotoxins such as MPTP and paraquat cause the elevation of H3 and H4 acetylation.^212–215^ Dieldrin, a neurotoxin associated with PD, increased acetylation levels at H3 and H4 as well as HAT CBP protein levels.^216^ In addition, anacardic acid, one of the HAT inhibitors, reduced dieldrin-mediated dopaminergic neuron death.^217^ Rotenone decreased the SIRT1 level and promoted the p53 expression through increased H3K9 acetylation on the p53 gene promoter, which induced neuronal cells.^218^ Aberrant upregulation of histone acetylation at H2A, H3, and H4 and simultaneous reduction of multiple HDACs were observed in 1-methyl-4-phenylpyridinium ion (MPP^+^)-treated cells and MPTP-treated mouse brains as well as midbrain tissues from human PD patients. Garcinol, another HAT inhibitor, repressed, but several HDAC inhibitors such as MS-275 and TSA potentiate MPP+ induced cell death.^219^ These results suggest that PD-related environmental toxins cause increased histone acetylation through CBP upregulation or HDAC downregulation (Fig. 6). For example, H3K9ac is increased in the substantia nigra pars compacta in brain samples from PD patients compared to brain samples from age-matched controls.^220^ Recent next-generation sequencing data have shown that H3K27 hyperacetylation dysregulates gene expression in PD. Toker et al. suggested that H3K27 acetylation is uncoupled from transcription in the PD brain.^221^ Using prefrontal cortex tissue from two cohorts of idiopathic individuals with PD and control, the global histone acetylation was increased at several histone residues in PD. The elevation of H3K27ac was prominent. ChIP-seq analysis revealed that genome-wide H3K27 hyperacetylation regions harbor PD-related genes such as *SNCA*, *MAPT*, *APP*, *PRKN*, and *PARK7*. Specifically, they also revealed that H3K27 hyperacetylation regions are associated with p300 binding sites obtained from relevant ChIP-seq data. The integration of the ChIP-seq data with the RNA-seq data shows that the correlation between H3K27ac and transcription in the PD group is reduced in contrast to a positive correlation between promoter H3K27ac and transcription in the control group. In contrast, Huang et al. have shown that H3K27 acetylation is elevated in rotenone-treated dopaminergic N27 cells and even in the substantia nigra of human PD. Their ChIP-seq and RNA-seq analyses demonstrated that rotenone-mediated mitochondrial dysfunction induces H3K27 hyperacetylation, increasing gene expression and activating neuronal apoptosis.^222^

**Fig. 7:**
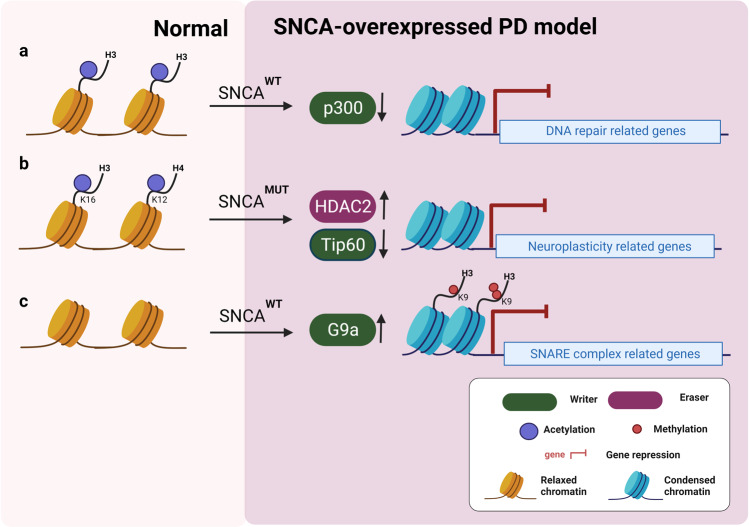
Effect of α-synuclein overexpression on PD-related gene expression. **a** Overexpression of wild-type α-synuclein (SNCA^WT^) reduces H3 acetylation levels by suppressing p300 expression, resulting in downregulating key genes related to DNA repair. **b** Mutant α-synuclein (SNCA^MUT^) expression induces HDAC2 expression but inhibits Tip60 expression, leading to the decrease of H3K16ac and H4K12ac at the neuroplasticity related genes. **c** Wild-type α-synuclein overexpression increases G9a expression, which negatively regulated SNARE complex gene expression (i.e., LICAM and SNAP25) by depositing H3K9me1/2

Several studies show the role of α-synuclein in altering histone acetylation (Fig. 7). α-Synuclein can cause neurotoxicity by reducing histone acetylation. The HDAC inhibitors sodium butyrate (NaB) and SAHA protect against α-synuclein-mediated neurotoxicity in both cellular and transgenic Drosophila models.^176,223,224^ Overexpression of α-synuclein in the dopaminergic neuronal cell line induced extensive transcriptional alteration, including a significant downregulation of key genes involved in DNA repair (Fig. 7a). Increased α-synuclein expression induces DNA damage and reduces histone H3 acetylation, which is rescued by HDACi NaB.^225–227^ Overexpressed nuclear α-synuclein binds to the gene promoter region of the peroxisome proliferator-activated receptor γ coactivator-1α (PGC-1α) and reduces PGC1 alpha expression, concomitantly with decreased histone acetylation.^228^ α-Synuclein reduces the p300 level and its HAT activity, which could reduce histone acetylation in the dopaminergic neuronal cell line.^229^

A well-characterized early-stage PD Drosophila model expressing a mutated form of human α-synuclein (SNCA^A30P^) shows a reduced level of Tip60 and an increased level of HDAC2. Furthermore, the alteration of Tip60/HDAC2 binding patterns and histone acetylation reduction such as H4K16ac and H4K12ac in common Tip60 target neuroplasticity genes result in concomitant epigenetic repression of these genes (Fig. 7b). Increasing Tip60 HAT levels in the Drosophila brain improves locomotion and short-term memory function deficits in the PD model. These results suggest that α-synuclein-related PD is involved in reducing histone acetylation by Tip60, suppressing Tip60 target neuroplasticity genes.^230^

### Dysregulation of histone methylation in Parkinson’s disease

6-OHDA, a PD-related toxin, reduces H3K4me3 and H3K27me3 in SH-SY5Y neuroblastoma cells. GSK-J4, a histone demethylases inhibitor, rescues the alteration of H3K4me3 and H3K27me3 as well as dopaminergic neuron loss and motor defects in 6-OHDA-induced PD rats. These data suggest that histone methylation dysregulation plays a critical role in PD pathology.^231^

Histone methylation is involved in the regulation of *SNCA* expression (Fig. 6). A recent study showed that H3K4me3 at the *SNCA* promoter was prominently elevated in brain samples from PD patients compared to controls. The deletion of H3K4me3 using locus-specific editing reduces a-synuclein in the neuronal cell line SH-SY5Y and idiopathic PD-iPSC-derived dopaminergic neurons.^232^

Overexpression of α-synuclein in transgenic Drosophila and inducible SH-SY5Y neuroblastoma cells causes an elevation of histone H3K9 methylations such as H3K9me1 and H3K9me2, repressive marks. The transient increase in α-synuclein in SH-SY5Y cells induces mRNA induction of the EHMT2 (G9a), which suppresses the expression of *L1CAM* (the neural cell adhesion molecule L1) and *SNAP25* (the synaptosomal-associated protein) by H3K9me1/2 deposition (Fig. 7c).^186,233^

## Histone modification in neuropsychiatric disorders

### Schizophrenia

Schizophrenia (SZ) is a chronic and complex psychiatric disorder which affects ~1% of the global population. It is characterized by positive symptoms (hallucinations, delusions, and disorganized behavior), negative symptoms (social withdrawal, decreased motivation, and diminished emotional expression), and cognitive impairment.^234–236^ Although the underlying neurobiology of SZ still remains to be clarified, genetic factors and environmental factors play key roles in the etiology of SZ.^237,238^ In addition, a growing body of evidence suggested that epigenetic dysregulations of genes related to neurotransmission, neurodevelopment, and immune function are linked to SZ.^239^

### Dysregulation of histone acetylation in schizophrenia

**Fig. 8:**
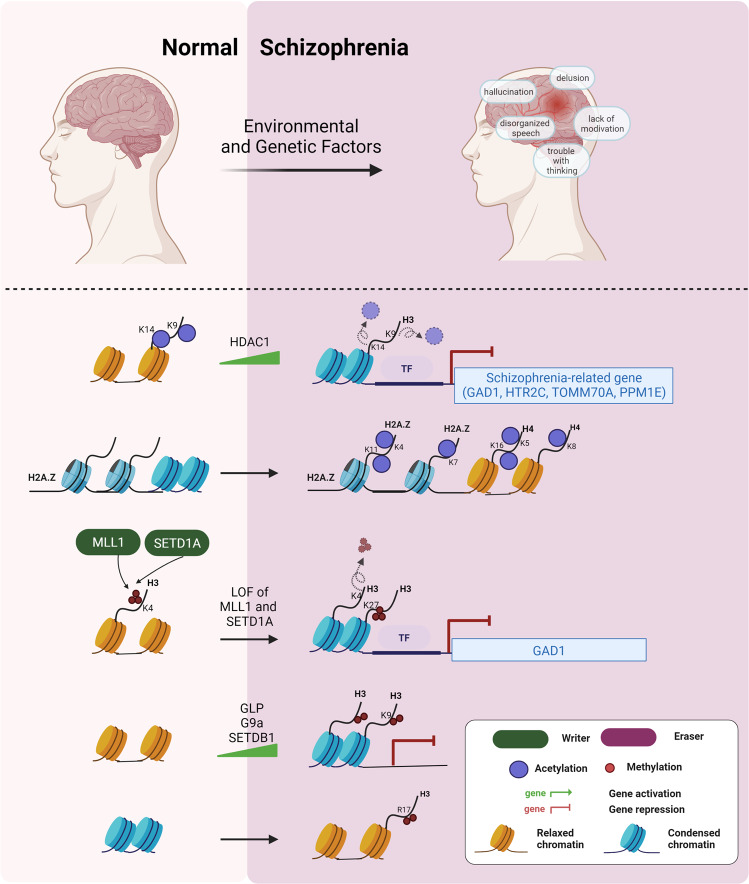
Dysregulation of histone-modifying enzymes and histone modifications in schizophrenia (SZ). Increase of repressive histone marks (e.g, H3 deacetylation by HDAC1, decrease of H3K4me3 by loss of MLL1 or SETD1A mutation, and increase of H3K27me3) suppresses GAD1 expression. Increase in H2A.Z acetylation, H3K9me3, and H3R17me is observed in SZ

Altered expression of neurotransmission-related genes from gamma-aminobutyric acid-ergic (GABAergic) and serotonergic neurons are often associated with SZ. Tang et al. found that mRNA expressions of a set of schizophrenia-related genes (*GAD1*, *HTR2C*, *TOMM70A*, and *PPM1E*) were reduced in prefrontal cortex (PFC) sample from SZ patient (postmortem human brain). Concomitantly, H3K9ac and H3K14ac levels in the promoter regions of those genes were decreased.^240^ Another study revealed that HDAC1 expression is increased in the PFC of SZ. Furthermore, GAD1(GAD67) expression is negatively correlated with the expression of HDAC1.^241^ These results suggested that histone acetylation reduction in neurotransmission-related genes have been mostly implicated in SZ (Fig. 8).

In contrast, Farrelly et al. observed that both NPC and 4-week-old forebrain neuron derived from SZ have a combinatorial hyperacetylation of H2A.Z (H2A.ZK4acK7acK11ac) and H4 (H4K5acK8acK16ac). These results were confirmed in postmortem human brain of SZ cases. Mechanistically, BRD4 recognizes combinatorial H2A.Z acetylation, leading to aberrant expression of genes related to neurodevelopmental components of SZ. JQ1, BET bromodomain inhibitor, ameliorates transcriptional abnormality in SZ-derived neurons.^242^

### Dysregulation of histone methylation in schizophrenia

Histone methylation and its modifying enzymes were differentially regulated in SZ (Fig. 8). Huang et al. revealed that GABAergic mRNA expression plays key roles in maturation of PFC, and GABAergic gene expression correlates with the increased H3K4me3 levels in the promoter region. In postmortem PFC from SZ subjects, H3K4me3 is decreased while H3K27me3 is increased in the *GAD1* promoters. They also found that MLL1-mediated H3K4me3 is required for GAD1 expression.^243^ Recent genome-wide studies reported that genes involved in H3K4 methylation are associated with SZ, and that loss of function (LOF) mutations in SED1A is a substantial risk to SZ.^244–247^ Further studies identified that Setd1a^+/−^ mice mimicking mutation of SZ patient or LOF display SZ-related phenotype.^248–250^ Mukai et al. found that SETD1A targets are highly expressed in pyramidal neurons. SETD1A is recruited by cortex-specific activators at enhancers while more generally expressed activators recruit SETD1A to promoters. MEF2 and SETD1A are co-localized at enhancer. Interestingly, promoter-bound SETD1A activates gene expression, but enhancer-associated setd1a inhibits MEF2 transactivity by methylating MEF2. Restoring SETD1A expression in adulthood rescues cognitive impairment in Setd1a deficient mice.^251^ These results suggested that MLL1 or SETD1A is a novel therapeutic target for SZ.

*GLP*, *G9a*, and *SETDB1* mRNAs are increased in postmortem PFC from SZ subjects. Consequently, H3K9me2 levels are increased in SZ.^252^ Another study, on the other hand, showed that high levels of H3R17 methylation (active mark) are found in PFC of subject with SZ.^253^ Recently, Girdhar et al. profiled cell-type specific histone modifications from PFC with SZ. Interestingly, ChIP-seq using H3K4me3 and H3K27ac shows differential epigenome signatures between neuron and non-neuron: H3K27ac, large genome coverage (20%) in neuron chromatin versus 15–16% coverage in non-neuron chromatin. The extended H3K27ac coverage in neuron includes intron and intergenic region, suggesting that cell-type specific signatures at regulatory and disease-associated non-coding sequences play critical roles in SZ.^254^

### Major depressive disorder

Major depressive disorder (MDD) is a chronic and debilitating illness that affects approximately 6% of the global population each year.^255^ It is characterized by a wide spectrum of symptoms including depressed mood, disturbed sleep and appetite, diminished ability to think or concentrate, excessive guilt, and recurrent suicidal thoughts, which overlap with depressive symptoms in schizophrenia and bipolar disorder.^256^ MDD is associated with multi brain regions including PFC, hippocampus, amygdala, and nucleus accumbens (NAc). These regions are connected structurally and functionally as a circuit (cortical-striatal-limbic circuit). In many case, significant structural and functional alterations in these brain regions and circuit are involved in MDD.

The heritability of MDD has been estimated at about 35% based on the family and twin-based studies.^257^ Additionally, environmental factors, such as prenatal factors, stress and drug abuse are associated with developing MDD. Recently, childhood events such as sexual, physical or emotional abuse have been reported to be strongly associated with the risk or the severity of MDD. On the other hand, protective environmental factors such as social and emotional supports and exercise can prevent MDD.^256^ Interestingly, recent studies have focused on epigenetic regulation by environmental factors of early life experiences, which may explain an individual difference in vulnerability and resilience to stress.^258^

### Dysregulation of histone acetylation in major depressive disorder

**Fig. 9:**
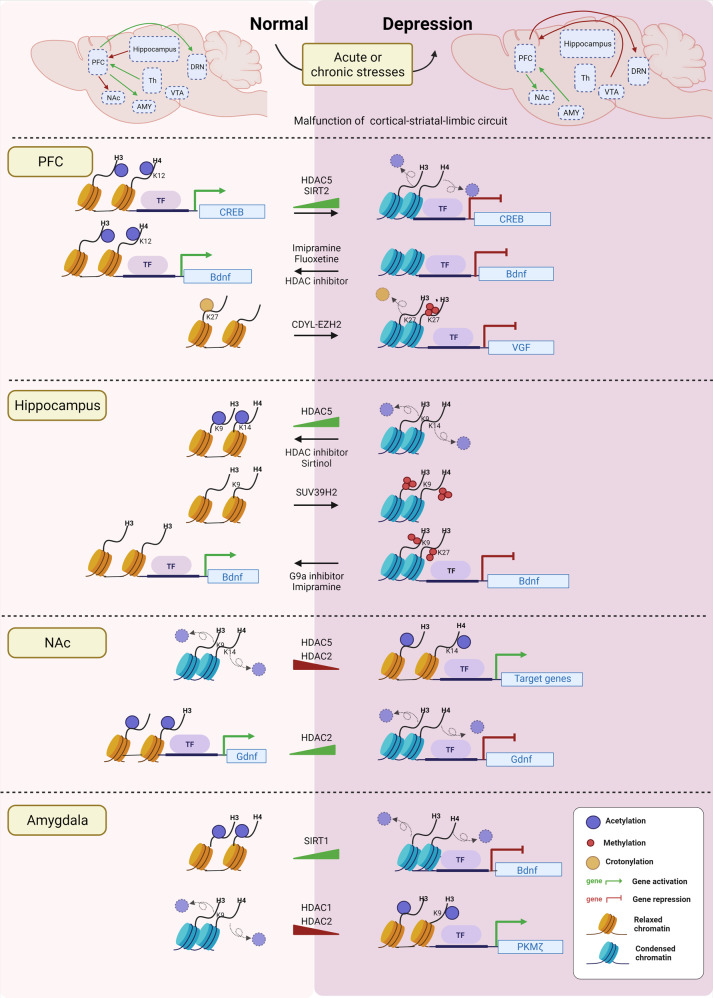
Dysregulation of histone-modifying enzymes and histone modifications in major depressive disorder (MDD). Neural circuit (cortical-striatal-limbic circuit) is structurally functionally disconnected in MDD. Histone modifications in brain regions such as prefrontal cortex (PFC), hippocampus, nucleus accumbens (NAc), and amygdala are various. In general, repressive marks are increased in PFC and hippocampus. In NAc and amygdala, active or repressive histone marks are observed in the promoter regions

Alteration in histone acetylation under active (e.g., defeat stress) or passive (e.g., social isolation) stress could contribute to the pathogenesis of MDD (Fig. 9). PFC is a key brain region responsible for cognitive information and is closely related to MDD. A recent study showed that chronic social defeat stress-induced *Hdac5* and *Sirt2* mRNA expression in PFC of mice. This caused a decrease in H3 and H4 acetylation, resulting in CREB gene repression.^259^ Treatment with HDAC inhibitors and/or antidepressant drug (e.g., imipramine, fluoxetine) significantly elevated H4K12ac level at the *Bdnf* promoter in the PFC along with increased Bdnf expression.^259,260^ Additionally, the latest studies proposed that histone crotonylation play a key role in regulating stress-induced depression. Chronic social defeat stress lowers the level of histone crotonylation by upregulating chromodomain Y-like protein (CDYL), a crotonyl-Coenzyme A hydratase, in the PFC. Furthermore, gain-of-function and loss-of-function studies have shown that CDYL regulates stress-induced depressive behavior. Mechanistically, CDYL-EZH2 complex removes H3K27cr and adds H3K27me3 to the *VGF* promoter.^134^

Several studies showed the altered histone acetylation in the hippocampus under active stress. In hippocampus of chronic unpredictable stress-induced rat, H3K9ac and H4K12ac were reduced with increasing HDAC5 expression. HDAC5 inhibitor treatment relieved anxiety- and depression-like behaviors, showing that H3K9ac and H4K12ac levels were increased.^261^ Chronic social defeat stress in mice causes a transient increase but followed by a persistent decrease in the H3K14ac levels in hippocampus. These changes were reversed by fluoxetine.^262^ In addition, chronic variable stress (CVS) mimicking certain symptoms of depression in human induced H4K12ac reduction in hippocampus subregions (CA3 and dentate gyrus) of CVS animals. Administration of Sirtinol, a class III sirtuin inhibitor, selectively increased H4K12ac in CVS-treated hippocampus. SirT1 deacetylase activity is increased in CVS.^263^

Recent studies have shown that histone acetylation is dysregulated in NAc associated with reward processing under active stress. Chronic social defeat stress decreases HDAC5 functions in NAc, leading to increased histone acetylation and gene activation of HDAC5 target genes.^264^ Similarly, chronic social defeat stress decreased HDAC2 expression, which induced a transient decrease but a persistent increase in the H3K14ac levels in NAc.^265^ These results are opposite for PFC and hippocampus, suggesting that histone modification to chronic stress are differentially regulated according to brain regions. Glial cell-derived neurotrophic factor (Gdnf) is a growth factor for the survival and maintenance of the dopaminergic neurons. Uchida et al. found that Gdnf expression levels in NAc determines susceptibility and adaptation to chronic stress. Chronic ultra-mid stress enhanced HDAC2 expression, which decreases H3 acetylation at *Gdnf* promoter. The low expression of Gdnf is more susceptible to chronic stress, suggesting that epigenetic regulation of Gdnf contributes to behavior responses to chronic stress.^266^

Recent studies reported that stress regulation of histone acetylation in amygdala, responsible for processing emotional information, is related to MDD. SIRT1 expression is increased under chronic unpredictable mild stress. This causes the decreased *Bdnf* expression, showing depression-like behavior. SIRT1 depletion in mouse model and neuron reversed depression behavior.^267^ On the other hand, Guan et al. showed that early life stress enhanced H3K9ac along with the decrease in HDAC1 and HDAC2 in the amygdala. The enhanced H3 acetylation may stimulate synaptic plasticity-related gene (PKMζ), resulting in anxiety-like behavior.^268^

### Dysregulation of histone methylation in major depressive disorder

Increasing evidence suggested that repressive histone methylation is increased under MDD (Fig. 9). Post-traumatic stress or chronic unpredicted mild stress drives an induction of H3K9me2 at *Bdnf* promoter and a reduction of Bdnf expression in the PFC and hippocampus. G9a inhibitor relieved depression behavior with decreasing H3K9me2.^269,270^ Conversely, chronic social stress decreased G9a expression and H3K9me2 in NAc.^271^ These studies suggested that the differential levels of G9a-mediated H3K9me2 according to brain regions are associated with MDD. Recently, it is reported that acute stress-induced Suv39h2-mediated H3K9me3 enrichment at transposable element in hippocampus.^272^ In addition, chronic defeat stress increased H3K27me2 on *Bdnf* promoter in hippocampus, resulting in Bdnf downregulation. Imipramine treatment reduced H3K27me2 and conversely increased H3 acetylation along with HDAC5 downregulation.^273^

## Conclusions and future perspectives

In recent decades, many researchers have studied the underlying mechanisms that lead to neuronal development and disease. The processes of neurogenesis and neurodisease are influenced by various internal and external factors. During these processes, epigenetic marks, including histone variant exchange, histone modifications, and DNA methylation, are dynamically changed, thereby modulating the chromatin states. In this review, comprehensive information on the roles of histone modifications in neurogenesis and neurodisease is provided. Histone modifications such as acetylation and methylation are somewhat complex. These modifications are differentially implicated in neuron development and disease; some active or repressive marks globally increase or decrease, respectively, during these processes. In other cases, gene-specific changes in histone modifications modulate the expression of key genes related to neurogenesis and neurodisease. In addition, the spatiotemporal dynamics of histone modifications lead to the complexity of neurogenesis and neurodisease. Despite increased knowledge on these topics, many questions remain unclear regarding the mechanism of how these modifications regulate these processes. First, most of aforementioned neurodisease studies observed alteration of histone PTMs in postmortem subjects. Is alteration in histone PTM a driver or a passenger of neurological disease process? Second, various novel PTMs are identified. It would be interesting to investigate the role of new histone PTMs in neurogenesis and neurological disease. Third, extensive mechanistic studies are needed to determine which transcription factors (e.g., activator) cooperate with histone-modifying enzymes or which reader proteins are recruited to modified histones. Last, brain bulk brain samples have been used for genome-wide studies. Because brain tissues are composed of various cell types, genome-wide approach in single cell level may provide us more precise information. Several new techniques including next-generation sequencing at a single cell level, gene editing, and 3D brain organoids provide promising clues that may help us decipher these challenges.

Several HDAC inhibitors are preclinically evaluated for neurodegenerative and neuropsychiatric diseases. HDAC inhibitors have profound therapeutic potential with neuroprotective, neurotrophic, and antidepressant-like properties^265,274–283^ To date, there are only a few ongoing clinical trials for evaluating neurodegenerative disease due to safety concerns of HDAC inhibitors. The development of more specific and selective HDAC inhibitors would overcome the concerns associated with HDAC inhibitors. Indeed, a comprehensive map of dynamic epigenetic changes during neurogenesis and degeneration will help to develop innovative therapeutics for neurodegenerative and neuropsychiatric disorders.
